# A comparison of cognitive factors identified by Exploratory Factor Analysis of the original ADAS‐cognitive subscale and a novel extended version in mild cognitive impairment and Alzheimer’s dementia

**DOI:** 10.1002/alz.087044

**Published:** 2025-01-03

**Authors:** Mark S. Overby, Sean McGinity, Albert Botchway, Ronald F. Zec, Thomas A. Ala, Zachary Settelmyer, Eukesh Ranjit, Raj C. Shah, Erin R. Hascup, Mehul A. Trivedi

**Affiliations:** ^1^ SIU School of Medicine, Springfield, IL USA; ^2^ Rush University Medical Center, Chicago, IL USA; ^3^ Southern Illinois University School of Medicine, Springfield, IL USA

## Abstract

**Background:**

The original Alzheimer’s Disease Assessment Scale – Cognitive Subscale (oADAS) is one of the most common measures of cognition used in clinical trials of Alzheimer’s dementia (AD). Despite this, the oADAS has faced criticism for lacking sensitivity to Mild Cognitive Impairment (MCI), potentially due to ceiling effects for certain subsets. Many attempts have been made to improve the sensitivity of the oADAS to MCI by including additional subtests. We previously used exploratory factor analysis (EFA) on the subtests from the oADAS and a novel extended version of the ADAS (eADAS). We identified two factors for both the oADAS (general and praxis factors) and the eADAS (memory/speed and general factors). In the present study, we examined group differences between normal controls, MCI, and AD on the four factors previously identified.

**Method:**

This analysis included data from 680 normal controls above the age of 60 who were participants in the SIU Longitudinal Cognitive Aging Study (SIU LCAS), 63 individuals with amnestic MCI (MCI), and 51 individuals with possible/probable AD. Participants were administered the oADAS using standardized procedures along with an extended version (eADAS) that included additional measures of processing speed, language, learning/memory, and visuospatial skills. A 3‐group ANCOVA was completed between control, MCI, and AD groups and the four factor components. Age, gender, and education were included as covariates.

**Result:**

For the oADAS, a general factor was able to discriminate between the three groups (p’s<0.001) while a praxis factor showed differences between control and AD groups and between MCI and AD groups (p’s<0.001) but not between MCI and controls (p>.90). For the eADAS, both a memory/speed factor and a general factor were able to significantly discriminate between all three groups (p’s<0.05).

**Conclusion:**

This study demonstrated that most of the identified cognitive factors were able to discriminate between the three groups with the exception of the praxis factor from the oADAS, which did not demonstrate significant differences between the MCI and control groups. More research is needed to further determine which subtests from the oADAS and eADAS are most sensitive to cognitive changes in normal and abnormal cognitive aging.

